# Population-Based Limits of Urine Creatinine Excretion

**DOI:** 10.1016/j.ekir.2022.08.025

**Published:** 2022-08-31

**Authors:** Bryan Kestenbaum, Joachim H. Ix, Ron Gansevoort, Michael L. Granda, Stephan J.L. Bakker, Dion Groothof, Lyanne M. Kieneker, Andy N. Hoofnagle, Yan Chen, Ke Wang, Ronit Katz, David K. Prince

**Affiliations:** 1 Division of Nephrology, Kidney Research Institute, University of Washington, Seattle, Washington, USA; 2 Division of Nephrology, Department of Medicine, University of California, San Diego, California, USA; 3 Division of Nephrology, Department of Internal Medicine, University Medical Center Groningen, University of Groningen, Groningen, the Netherlands

**Keywords:** creatinine, urine collection

## Abstract

**Introduction:**

The validity of a timed urine collection is typically judged by measurement of urine creatinine excretion, but prevailing limits may be unreliable. We sought to empirically derive population-based limits of excretion for evaluating the validity of a timed urine collection.

**Methods:**

Covariate and 24-hour urine data were obtained from 3582 participants in the Chronic Renal Insufficiency Cohort (CRIC) study, 814 participants in the Modification of Diet in Renal Disease (MDRD) study, 1010 participants in the Jackson Heart Study (JHS), and 8536 participants in the Prevention of Renal Vascular End Stage Disease (PREVEND) study. Weight, height, age, sex, and serum creatinine concentrations were evaluated as potential predictors of urine creatinine excretion using Akaike Information Criteria, R-squared values, and deviance. Bias and precision of the fitted models were assessed by analyses of residuals. Agreement between 24-hour creatinine clearance and ^125^I-iothalamate clearance was assessed before and after exclusion of potentially invalid urine samples.

**Results:**

A best-fitting model to predict 24-hour urine creatinine excretion among the 9199 discovery cohort members included sex-specific terms for weight, height, and age (R-squared = 0.328). This model had a median bias of +4.3 mg creatinine/day (95% confidence interval −5.6, +13.3 mg/day) in 4599 validation cohort members, and 82% of observed values were within 30% of predicted model. Serum creatinine concentrations only marginally improved model precision but reduced bias in persons with advanced chronic kidney disease (CKD).

**Conclusion:**

The limits of urine creatinine excretion derived here represent the most valid and representative data for appraising the adequacy of a timed urine collection.

## Graphical abstract

**Figure:**
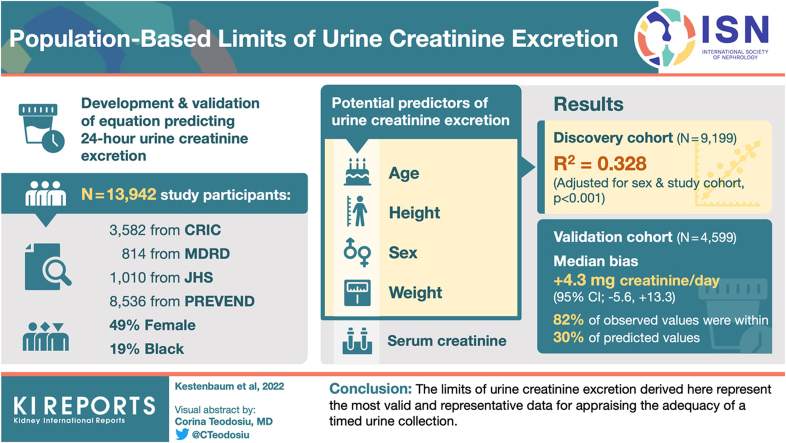

Timed urine collections have wide-ranging applications in clinical medicine and research, including quantification of urine albumin excretion, detection of monoclonal proteins, workup of specific endocrine disorders, and measurement of kidney stone precursors.^1^, ^2^, ^3^, ^4^ Timed urine collections are also used to calculate creatinine clearance as a means of estimating the glomerular filtration rate (GFR), particularly in persons with advanced CKD.^5^^,^^6^ A major barrier to interpreting the results obtained from a timed urine sample is the potential for error in the collection process, which often occurs outside the clinic or hospital setting. Potential errors in urine collection times are typically addressed by simultaneous measurement of urine creatinine excretion under the assumption that creatinine is produced at a constant rate by skeletal muscle under steady-state conditions.^7^

Prevailing weight and sex-based limits of creatinine excretion used to judge the adequacy of a timed urine sample (e.g., 15 to 20 mg/kg per day in women and 20 to 25 mg/kg per day in men) may be unreliable. Studies to derive these limits lacked adequate sample size to reliably estimate lower and upper limits of creatinine excretion and included mostly white individuals despite potential differences in skeletal muscle mass by race or ethnicity.^8^, ^9^, ^10^, ^11^, ^12^, ^13^, ^14^, ^15^ Most prior studies are further limited by a relative paucity of persons with advanced CKD for whom serum creatinine-based estimation of GFR may be less reliable.^16^^,^^17^

The goal of this study was to empirically derive and validate equations to predict lower and upper limits of urine creatinine excretion for evaluating the validity of a timed urine sample. To accomplish this goal, we analyzed 24-hour urine creatinine, anthropometric, demographic, and laboratory data from more than 13,000 participants in 4 cohort studies that included persons with and without kidney disease. We indirectly evaluated the utility of the derived limits by comparing the agreement between 24-hour creatinine clearance and ^125^I-iothalamate clearance in a subset of participants before and after exclusion of potentially invalid samples that were outside the derived cutoff values.

## Methods

### Study Populations

We used data from 3582 participants in the CRIC study, 814 participants in the MDRD study, 1010 participants in the JHS, and 8536 participants in the PREVEND study who had 24-hour urine creatinine measurements available for analysis. The CRIC study is a multicenter US cohort study of 3939 patients with CKD; 3582 participants completed a 24-hour urine collection at baseline.^18^^,^^19^ Exclusion criteria in CRIC included polycystic kidney disease, kidney transplantation, HIV disease, immunosuppression, multiple myeloma, and severe heart failure. The MDRD study was a cooperative randomized clinical trial of dietary protein restriction and blood pressure reduction among 840 patients 18 to 70 years of age with known CKD.^20^ Persons with insulin-requiring diabetes mellitus or a body weight less than 80% or greater than 160% of predicted were excluded, and 24-hour urine samples were collected at the baseline visit following the run-in procedure and before randomization. The JHS is a community-based study of cardiovascular disease among 5302 African American adults residing in the Jackson, Mississippi metropolitan area.^21^ A randomly selected subset of 1028 JHS participants provided a 24-hour urine sample within 1 week of the baseline exam, and collections were returned to the examination center for recording of total urine volume.^22^ The PREVEND study is a prospective study of microalbuminuria and its associations with cardiovascular disease in persons 28 to 75 years of age residing in the city of Groningen (The Netherlands).^23^ Persons with insulin-dependent diabetes mellitus were excluded. PREVEND study participants collected 2 consecutive 24-hour urine samples during 2 visits, separated by 3 weeks, to an outpatient clinic; we used the first collection for these analyses. Efforts to ensure complete 24-hour urine collections are described in Supplementary Table S2.

Beginning with a total analytic sample of 13,942, we excluded 100 individuals who were missing information on height or weight, 25 individuals who had extreme values for height (< 140 or >200 cm), and 19 individuals who had extreme values for weight (<40 or >180 kg), leaving a final analytic sample of 13,798, which served as the study population. For models that included serum creatinine concentrations, we further excluded 75 individuals who were missing creatinine data and 9 individuals who had extreme values (>7 mg/dl).

### Measurements

Urine creatinine concentrations were measured using the Jaffe method on the Roche diagnostics platform in the CRIC study, a kinetic alkaline picrate assay on a Beckman Astra-8 platform (Beckman Instruments, Irvine, CA) in the MDRD study, a Vitros 950 or 250 analyzer (Ortho-Clinical Diagnostics, Raritan, NJ) in the JHS study, and an enzymatic method on a Roche Modular analyzer, using reagents and calibrators from Roche (Roche Diagnostics, Mannheim, Germany) in PREVEND.^23^, ^24^, ^25^ CRIC and JHS urine samples were recalibrated as described in Supplementary Table S1. We adjusted models for individual study cohort with CRIC as the reference group. We estimated GFR from age, sex, self-reported race, and serum creatinine concentrations using the 2009 CKD Epidemiology Collaboration equation.^26^ We calculated creatinine clearance as:

Creatinine clearance (ml/min) = (Urine creatinine × Urine volume)/(Serum creatinine)Where, urine and serum creatinine are expressed in mg/dl and urine volume is expressed in ml/min.

All MDRD study participants and a randomly selected one-third of CRIC study participants completed ^125^I-iothalamate clearance measurements for determination of GFR (iGFR). Trained personnel in each study administered ^125^I-iothalamate and collected plasma and timed urine samples during 4 subsequent collection periods. In CRIC, the first collection period was excluded from the analyses. iGFR was calculated as the weighted average of iothalamate clearance during the collection. To facilitate comparison with creatinine clearance, we did not standardize iGFR to body surface area.

### Analysis

We randomly selected two-thirds of the study population to serve as the discovery cohort and one-third to serve as the validation cohort, stratified by categories of estimated GFR (0–29, 30–44, 45–59, 60–89 and ≥90 ml/min per 1.73 m^2^). Graphical assessment of 24-hour urine creatinine excretion values indicated a unimodal distribution with a right-sided tail. Scatterplots of urine creatinine excretion and the continuous predictors showed an increasing mean-variance relationship. To best capture the distribution of errors for this characteristic, we implemented a Gamma generalized linear model with log link, which yields wider prediction intervals for greater predicted mean urine creatinine values. This models differences between recalibrated CRIC study urine creatinine values and the other cohorts on a multiplicative rather than additive scale.

Primary predictor variables were sex, weight (kg), height (cm), and age (years). We explored whether serum creatinine concentrations or self-reported race could improve performance of the anthropometric and demographic model. All models included dummy variables for sex and individual study cohort (MDRD, JHS, PREVEND) with recalibrated CRIC urine creatinine values serving as the reference group.

We investigated potential nonlinear relationships using polynomial, logarithmic, and inverse transformations. We tested for potential interactions among the demographic and anthropometric characteristics. Sex-specific differences in the associations of weight and age with 24-hour urine creatinine excretion motivated construction of separate models among men and women. For models that included serum creatinine concentrations, we modeled this covariate using natural splines with knots at 0.7 mg/dl in women and 0.9 mg/dl in men at the median and 75^th^ percentiles of each sex, based on empirical associations with urine creatinine excretion. We considered model performance to be improved by a decrease in the Aikake Information Criterion within sex-specific models with further consideration of changes in R-squared values, deviance, and graphical inspection of residual plots. We tested the precision and accuracy of the fitted models in the validation cohort using root mean squared errors, the median and interquartile range of residuals, and the proportion of observations within 30% of predicted model.

We computed prediction limits of 24-hour urine creatinine excretion using bootstrap-based prediction intervals based on covariates. We compared empirically derived model-based limits with prevailing sex and weight-based limits. To indirectly assess application of the prediction limits, we determined the agreement between 24-hour creatinine clearance and iGFR in a subset of participants before and after excluding urine samples with creatinine excretion values outside these limits. All analyses were conducted using Stata version 13.1 (StataCorp., College Station, TX) or R version 3.6.2 (R core team, Vienna, Austria).

## Results

### Study Population

Among 9199 participants in the discovery cohort, the mean age was 52 ± 13 years; 4532 (49%) were women; and 1748 (19%) self-reported their race as Black (Table 1). The mean 24-hour urine creatinine excretion in the discovery cohort was 1338 ± 466 mg (by subgroups shown in Table 2). Estimated GFR was ≥ 60 ml/min per 1.73 m^2^ in 67% of discovery cohort members, 30 to 59 ml/min per 1.73 m^2^ in 24%, and <30ml/min per 1.73 m^2^ in 9%. Participant characteristics were similar in the discovery cohort and the validation cohort. Among 8515 participants in the PREVEND study who repeated their 24-hour urine collection an average of 3 weeks later, the median interindividual difference in urine creatinine excretion was 11% (interquartile range 5%, 22%).

**Table 1:** Baseline characteristics of the discovery and validation cohorts

Characteristic		Discovery cohort (*n* = 9199)	Validation cohort (*n* = 4599)
Age, yr	Mean (SD)	52 (13)	52 (13)
	Range	19, 85	21, 82
Women		4532 (49.3)	2289 (49.8)
Self-reported Black race		1748 (19.0)	882 (19.2)
Weight, kg	Mean (SD)	82 (18)	82 (18)
	Range	40, 180	41, 180
Height, cm	Mean (SD)	171 (10)	171 (10)
	Range	140, 200	140, 200
Estimated glomerular filtration rate			
≥ 60 ml/min/1.73 m^2^		6187 (67.7)	3094 (67.7)
30–59 ml/min/1.73 m^2^		2160 (23.6)	1079 (23.6)
<30 ml/min/1.73 m^2^		796 (8.7)	398 (8.7)
Serum creatinine, mg/dl^a^^,^^c^	Mean (SD)	1.3 (0.6)	1.3 (0.6)
Urine creatinine excretion (mg/d)^b^	Mean (SD)	1338 (466)	1345 (460)
	Range	43, 5175	45, 5125
Urine albumin excretion			
≥30 mg/d		6308 (73.0)	3119 (72.5)
<30 mg/d		2337 (27.0)	1186 (27.5)
Study cohort			
CRIC		2382 (25.9)	1169 (25.4)
MDRD		528 (5.7)	284 (6.2)
JHS		669 (7.3)	341 (7.4)
PREVEND		5620 (61.1)	2805 (61.0)

CRIC, Chronic Renal Insufficiency Cohort study; JHS, Jackson Heart Study; MDRD, Modification of Diet in Renal Disease study; PREVEND, prevention of renal and vascular end stage disease study.

All values in table expressed as mean (SD), n (%) or minimum, maximum.

a To convert serum creatinine from mg/dl to umol/l multiply by 88.4.

b To convert urine creatinine from mg/d to mmol/d divide by 113.12.

C *n* = 9143 in discovery cohort and 4571 in validation cohort with serum creatinine measurements.

**Table 2:** 24-hour urine creatinine excretion in the discovery cohort (*n* = 9199)

Characteristic	Women (n = 4532)	Men (*n*= 4667)
Overall	1102 (1092, 1111)	1568 (1555, 1581)
Age, yr		
<40	1195 (1176, 1214)	1666 (1635, 1697)
40–55	1137 (1122, 1153)	1651 (1627, 1675)
55–70	1045 (1028, 1062)	1499 (1480, 1519)
>70	929 (898, 960)	1368 (1327, 1408)
Self-reported race		
Non-Black	1105 (1095, 1115)	1574 (1561, 1588)
Black	1091 (1066, 1116)	1532 (1491, 1573)
Weight, kg		
<60	936 (915, 956)	1055 (969, 1142)
60–75	1071 (1059, 1084)	1359 (1332, 1386)
75–90	1137 (1119, 1154)	1536 (1518, 1553)
>90	1231 (1202, 1260)	1728 (1704, 1752)
Estimated glomerular filtration rate		
≥ 60 ml/min/1.73 m^2^	1126 (1116, 1137)	1622 (1607, 1637)
30–59 ml/min/1.73 m^2^	1058 (1032, 1084)	1503 (1474, 1532)
<30 ml/min/1.73 m^2^	997 (959, 1035)	1386 (1341, 1432)

All values in table expressed as mean (95% confidence interval).

### Development of Prediction Models

In univariate analyses, sex was the strongest single predictor of 24-hour urine creatinine excretion based on the reduction in Aikake Information Criterion, followed by weight, height, and age. The functional relationships of weight and age with 24-hour urine creatinine excretion differed by sex, motivating construction of separate models in men and women. The functional forms selected are displayed in Supplementary Figure S1. In models that included sex-specific terms for only weight, analogous to current clinical assessment, the adjusted R-squared value was 0.275 (Table 3). The addition of best-fitting terms for height and age progressively reduced the Aikake Information Criterion and deviance and increased the R-squared value. No meaningful improvement in prediction was achieved by addition of a product term for weight and height or by the inclusion of self-reported Black race. A final anthropometric and demographic model that included sex-specific terms for weight, height, age, and individual study cohort had an R-squared value of 0.328.

**Table 3:** Prediction of 24-hour urine creatinine excretion in the discovery cohort (*n* = 9199)

Model	AIC, women	AIC, men	Adjusted R-squared	Deviance	*P*-value
Adjustment for sex and study cohort	65,070	70,573	0.205	849	<0.001
Add weight (w)^a^	64,504	69,841	0.275	764	<0.001
Add height (h)^a^	64,282	69,786	0.309	717	<0.001
Add age (a)^a^^,^^b^	64,068	69,661	0.328	691	<0.001
Add height × weight interaction term	64,068	69,663	0.329	691	0.336
Add serum creatinine concentration (s)^c^^,^^d^	63,606	69,137	0.336	672	<0.001

AIC, Akaike Information Criteria.

a Selected functional forms are inverse of weight for both sexes; inverse of height squared for both sexes; age squared for women and age cubed for men.

b Final demographic and anthropometric model (model 1). Formulas are: Females: log(E[creatinine]) = 8.1–35.1∗w^−1^–14,526∗h^−2^–0.5∗a^2^. Males: log(E[creatinine]) = 8.2–54.1∗w^−1^–7885∗h^−2^–0.4∗a^3^.

c Serum creatinine concentrations modeled as natural splines with knots at 0.7, the median and the 75^th^ percentile for women and knots at 0.9, the median and the 75^th^ percentile for men.

d Final demographic, anthropometric, and serum creatinine model (model 2; *n* = 9143 participants with nonmissing serum creatinine values).

Higher serum creatinine concentrations were associated with progressively greater urine creatinine excretion up to threshold values of approximately 0.7 mg/dl and 0.9 mg/dl in women and men, respectively (Supplementary Figure S2). Thereafter, higher serum creatinine concentrations were associated with progressively lower urine creatinine excretion. The addition of serum creatinine modeled as a natural spline to fit these thresholds yielded small gains in prediction (Table 3).

**Figure 1:**
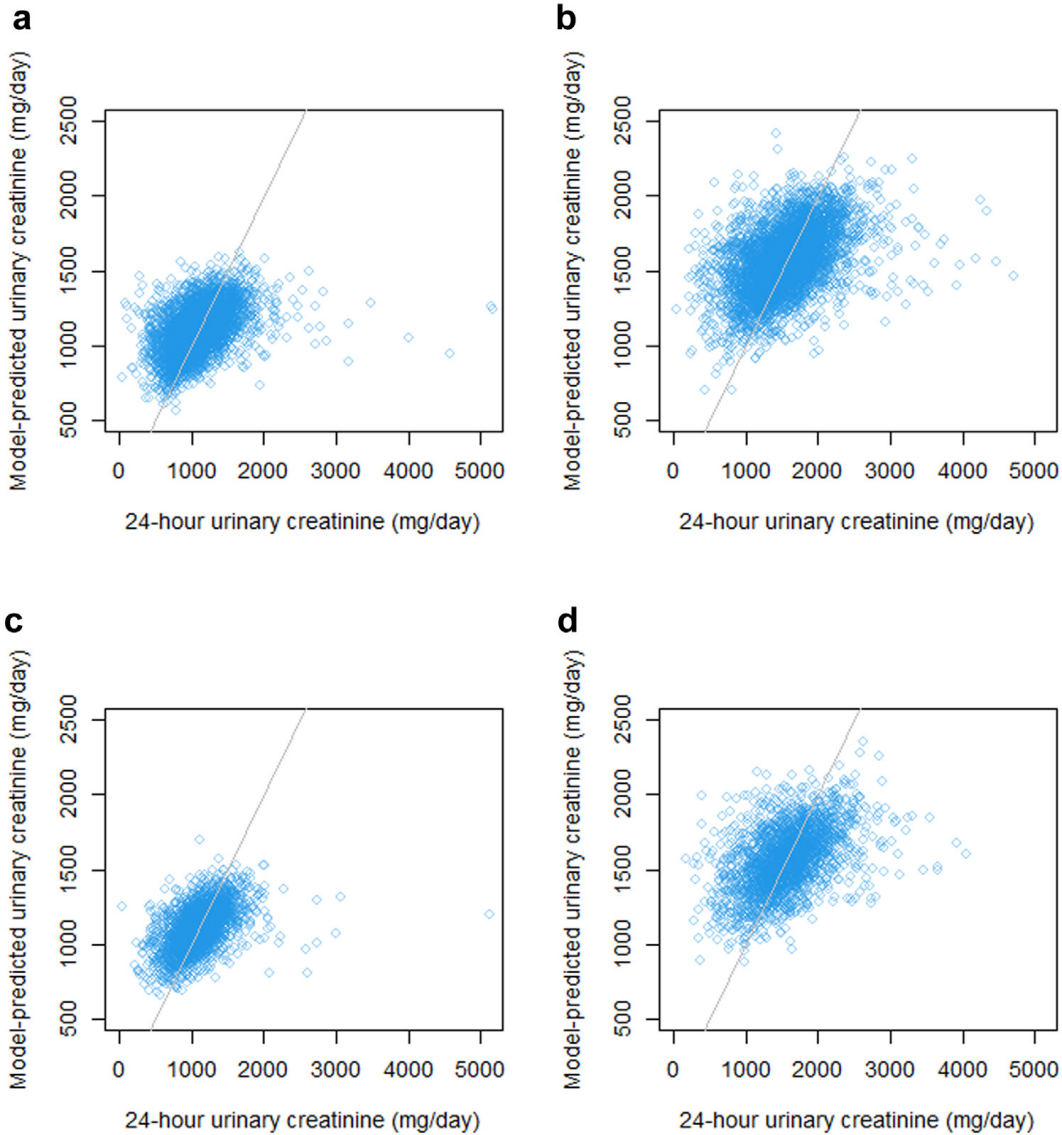
Observed versus predicted values of 24-hour urine creatinine excretion in the discovery for (a) women and (b) men and in replication cohort for (c) women and (d) men.

### Validation of Prediction Models

Among 4599 validation cohort members, the anthropometric and demographic model had a root mean squared error of 353 mg creatinine/day and median bias of +4.3 mg creatinine/day (95% confidence interval −5.6, +13.3 mg creatinine/day). 81.8% of the observed 24-hour urine creatinine measurements in the replication cohort were within 30% of those predicted by the model. Similar prediction statistics were observed for the anthropometric, demographic, and serum creatinine model as follows: root mean squared error of 345 mg creatinine/day, median bias of +1.9 mg creatinine/day (95% confidence interval −7.3, +11.8 mg creatinine/day), and 81.7% of observed measurements within 30% of predicted model.

The median difference between observed and predicted model urine creatinine excretion in the replication cohort was low among men and women (Figure 1) and among participants who self-reported their race as Black or non-Black, indicating low bias by sex and race (Table 4). Nevertheless, the anthropometric and demographic model systematically overestimated urine creatinine excretion among persons with low estimated GFR (median difference −82 mg creatinine/day for estimated GFR <30 ml/min per 1.73 m^2^). This bias was reduced using the model that included serum creatinine concentrations.

**Table 4:** Median bias and interquartile range among subgroups in the validation cohort

Characteristic	Anthropometric and demographic model (*n* = 4599)		Anthropometric, demographic, and serum creatinine model (*n* = 4571)	
	Median bias, mg/d (95% CI)	Interquartile range, mg/d	Median bias, mg/d (95% CI)	Interquartile range, mg/d
Women	+3.9 (−7.7, +15.5)	309.4	−0.3 (−10.4, +13.2)	305.9
Men	+5.5 (−10.7, +20.8)	463.3	+4.0 (−14.7, +20.7)	452.9
Black	+7.3 (−24.4, +33.3)	466.0	+12.3 (−22.6, +47.1)	458.4
Non-Black	+3.8 (−6.7, +13.4)	356.0	−0.1 (−9.3, +10.7)	347.0
estimated glomerular filtration rate (ml/min/1.73 m^2^)				
>60	+12.4 (+3.5, +23.4)	347.6	+11.4 (−1.7, +25.0)	338.8
30–60	−5.4 (−26.5, +31.4)	428.5	−13.0 (−46.9, +7.4)	424.2
<30	−81.5 (−124.2, −40.6)	420.6	−28.7 (−76.7, +1.9)	414.2
24-hour urine albumin				
<30 mg/d	−25.5 (−46.0, −4.8)	449.6	−27.6 (−44.9, −2.6)	458.8
≥30 mg/d	+12.6 (+2.4, +24.0)	352.1	+10.0 (−3.2, +25.0)	342.5

CI, confidence interval.

### Derivation and Indirect Evaluation of Model-Derived Prediction Limits

Applying clinical sex and weight-based limits to the replication cohort of 20 to 25 mg creatinine/kg per day in men or 15 to 20 mg creatinine/kg per day in women excluded 67% of the 24-hour urine samples. More conservative limits of 14 to 26 mg creatinine/kg per day in men or 11 to 20 mg creatinine/kg per day in women excluded 27% of the samples. Prediction intervals derived from model-based covariates were constructed to include 92.5% of replication cohort members (Table 5). For example, a 50-year old woman who is 160 cm tall and weighs 65 kg would have a predicted 5% lower limit of 588 mg creatinine/day.

**Table 5:** Predicted lower 5^th^ and upper 97.5^th^ percentile of 24-hour urine creatinine excretion

Women age 50 years old				
Weight in kg	Height in cm			
	150	160	170	180
55	487, 1296	532, 1386	568, 1505	602, 1586
65	543, 1443	588, 1561	625, 1652	665, 1751
75	577, 1546	627, 1671	675, 1818	711, 1876
85	621, 1629	670, 1758	703, 1871	756, 2020
95	643, 1716	699, 1824	750, 1969	785, 2083
105	663, 1749	726, 1901	769, 2015	803, 2145

Women age 60 yr old				
	Height in cm			
Weight in kg	150	160	170	180
55	465, 1220	500, 1325	535, 1418	567, 1513
65	509, 1356	555, 1489	586, 1557	619, 1660
75	555, 1439	593, 1588	641, 1685	668, 1774
85	582, 1539	626, 1662	668, 1785	711, 1868
95	611, 1601	660, 1744	706, 1857	735, 1967
105	633, 1672	688, 1807	717, 1909	766, 2029

Women age 70 yr old				
	Height in cm			
Weight in kg	150	160	170	180
55	437, 1149	466, 1245	504, 1329	525, 1399
65	485, 1260	519, 1362	550, 1463	588, 1552
75	516, 1362	563, 1466	599, 1567	626, 1651
85	548, 1424	591, 1544	626, 1657	667, 1753
95	567, 1506	613, 1615	651, 1713	691, 1834
105	588, 1560	642, 1684	684, 1791	715, 1889

Men age 50 yr old				
	Height in cm			
Weight in kg	160	170	180	190
65	682, 1799	704, 1855	721, 1911	738, 1966
75	750, 1996	792, 2065	806, 2131	823, 2194
85	823, 2200	860, 2254	873, 2323	914, 2371
95	883, 2338	916, 2413	939, 2504	962, 2542
105	933, 2461	968, 2547	993, 2641	1022, 2676
115	972, 2587	1004, 2653	1038, 2752	1060, 2834

Men age 60 yr old				
	Height in cm			
Weight in kg	160	170	180	190
65	652, 1719	676, 1796	696, 1849	715, 1861
75	725, 1922	751, 1995	779, 2069	799, 2096
85	798, 2098	823, 2153	842, 2253	867, 2273
95	852, 2226	892, 2319	896, 2367	931, 2461
105	901, 2365	929, 2449	952, 2509	981, 2570
115	938, 2505	972, 2568	990, 2621	1030, 2698

Men age 70 yr old				
	Height in cm			
Weight in kg	160	170	180	190
65	611, 1616	634, 1721	653, 1754	672, 1786
75	691, 1816	711, 1870	747, 1953	750, 2001
85	756, 1970	775, 2078	787, 2113	812, 2170
95	790, 2135	834, 2198	860, 2281	881, 2319
105	842, 2247	883, 2326	889, 2381	913, 2440
115	880, 2349	921, 2417	951, 2509	973, 2565

Iothalamate clearance measurements of GFR (iGFR) were available for 714 validation cohort members. (Supplementary Figure S3) The root mean squared error between 24-hour creatinine clearance and iGFR was 20.8 ml/min when all samples were included in the analysis (Table 6). The agreement between 24-hour creatinine clearance and iGFR improved upon excluding urine samples for which urine creatinine excretion was lower than model predicted limits, suggesting potential under-collection (Supplementary Figure S4). In contrast, the exclusion of potentially over-collected samples improved agreement for only the highest 2 to 3% of samples.

**Table 6:** Agreement between 24-hour creatinine clearance and iGFR in the replication cohort

Lower threshold	Upper threshold	Qualifying participants N (%)	RMSE (ml/min)	P30 (%)^a^
No exclusions		714 (100.0)	20.8	69.0
Model based exclusions^b^				
None	Upper 97.5%	690 (96.6)	16.7	70.4
None	Upper 95%	682 (95.5)	16.7	70.5
Lower 2.5%	Upper 97.5%	660 (92.4)	15.3	73.0
Lower 5%	Upper 97.5%	646 (90.5)	14.3	74.1
Lower 7.5%	Upper 97.5%	629 (88.1)	14.0	75.7
Lower 10%	Upper 97.5%	617 (86.4)	13.8	76.3
Lower 5%	Upper 95%	638 (89.4)	14.2	74.3
Lower 7.5%	Upper 95%	621 (87.0)	13.9	75.8
Existing limits				
15 mg/kg women	20 mg/kg women	182 (25.5)	14.6	74.2
20 mg/kg men	25 mg/kg men			

iGFR, glomerular filtration rate measured by ^125^I-iothalamate clearance; RMSE, root mean squared error.

a Proportion of values within 30% of predicted.

b Based on final anthropometric and demographic model (model 1).

## Discussion

Using data from 4 large cohort studies, including persons with and without kidney disease, we developed and validated models to predict lower and upper limits of urine creatinine excretion for assessing the validity of a timed urine sample. A model that included sex-specific terms for weight, height, and age had moderate precision and low bias except among persons with advanced CKD, who had systematically lower urine creatinine excretion than predicted. A second model that added serum creatinine concentrations reduced bias by low GFR. Importantly, the addition of self-described race to the models did not improve the prediction of urine creatinine excretion beyond that of sex, height, weight, and age. The agreement between 24-hour creatinine clearance and ^125^I-iothalamate clearance improved after excluding urine samples for which creatinine excretion was outside of model-based predicted limits. Applying prevailing sex and weight-based limits of 20 to 25mg creatinine/kg per day in men or 15 to 20 mg creatinine/kg per day in women to the 24-hour urine samples in this study excluded two-thirds of the collections. Given limitations of existing methods and strengths of the current study, we suggest that the empirical limits derived here represent the most valid and representative data for appraising the utility of a timed urine collection.

The measurement of creatinine in a timed urine sample is motivated by its theoretically stable production from creatine in skeletal muscle and nearly exclusive elimination by the kidneys.^27^^,^^28^ Under steady-state conditions, creatinine excretion in the urine should equal its production, which can be estimated from body size. Early equations to estimate urine creatinine excretion based on anthropometric characteristics were derived from small study populations with limited diversity and lacked a validation step.^29^^,^^30^ More recent studies have derived and validated equations to predict the mean 24-hour urine creatinine excretion values in larger cohorts.^9^, ^10^, ^11^ For example, a prediction model based on weight, age, and sex derived from 2466 individuals yielded similar bias and precision to those calculated here.^9^ Nevertheless, this study did not quantify upper and lower limits of creatinine excretion for assessing the adequacy of a timed urine sample.

There is no gold-standard method to definitively determine the accuracy of a timed urine collection, which often takes place outside of the clinic or hospital setting. Previous studies have investigated the utility of ingested para-aminobenzoic acid, which is rapidly excreted in the urine. Improvement in para-aminobenzoic acid recovery was reported after excluding 24-hour urine samples for which urine creatinine excretion was lower than sex and weight-based limits. Nevertheless, this approach is limited by use of an ingested compound and the ability to detect only under-collected samples. Given the inherent lack of a gold-standard, the adequacy of timed urine collections can be practically assessed using model-based population-derived limits, such as those determined here, similar to the approach used for many laboratory measurements. Such a strategy will represent a trade-off between accuracy on one hand and inclusiveness on the other and could be used to identify potential over-collections and under-collections. Based on the agreement between 24-hour creatinine clearance and direct measurements of GFR in this study, a reasonable balance was achieved by excluding the upper 2.5% and lower 5% of samples for a given sex, weight, height, and age.

Our study has several strengths. Discovery and validation analyses were conducted in a large and diverse study population that included persons with and without CKD. We calibrated urine creatinine measurements in the reference cohort to current isotope dilution mass-spectroscopy standards, promoting clinical application of the model-derived limits. We derived a second model specifically for individuals with advanced CKD or low muscle mass that included serum creatinine concentrations to account for potential bias that may occur in these individuals. An important limitation of this study is the residual variation in urine creatinine excretion after idealized modeling of sex, weight, height, and age, consistent with findings from previous studies. More comprehensive measurements of body composition using methods such as bioimpedance or imaging procedures could improve prediction but are impractical for clinical use. A second limitation is systematic overestimation of 24-hour urine creatinine excretion among persons with advanced CKD. This limitation was moderated by the addition of serum creatinine concentrations to the model. Limits derived from the serum creatinine model may be useful for evaluating timed urine samples in persons with advanced CKD. A third limitation is that comparisons between creatinine clearance and iGFR were limited to the CRIC and MDRD studies, which include only persons with established CKD.

In summary, we derived and validated prediction limits for 24-hour urine creatinine excretion in a large diverse study population based on readily obtainable anthropometric and demographic characteristics. Urine creatinine values from the derived equation are referenced to current laboratory standards. The application of model-derived prediction limits excluded far fewer 24-hour urine samples compared with accepted sex and weight-based limits. These considerations suggest application of the derived limits to assess the adequacy of timed urine collections in practice.

## Disclosure

BK reports personal fees from Reata Pharmaceutical outside of the submitted work. JI reports grants or contracts from the NIDDK and Baxter International, consulting fees from Bayer, Ardelyx, AstraZeneca, and Jnana Therapeutics, payment from American Society of Nephrology (ASN), support for travel and meetings from ASN and Kidney Disease: Improving Global Outcomes, participation in a Data Monitoring Committee for Sanifit International, and stocks from AlphaYoung. YC is an employee of analysis group. All the other authors declared no competing interests.

## Supplementary Material

Supplementary File (PDF)

**Figure S1.** 24-hour urine creatine excretion for women and men by weight, height and age in the discovery cohort.

**Figure S2.** Association of serum creatinine concentrations with 24-hour urine creatinine excretion in the discovery cohort for women and men.

**Figure S3.** 24-hour creatinine clearance and iothalamate clearance measurements of GFR among replication cohort members who completed iothalamate testing in the discovery and replication cohorts for women and men.

**Figure S4.** Agreement between 24-hour creatinine clearance and iothalamate clearance measurements of GFR among replication cohort members who completed iothalamate testing.

**Table S1.** Calibration of Jackson Heart Study and Chronic Renal Insufficiency Cohort study urine creatinine measurements to isotope dilution mass-spectroscopy standards.

**Table S2.** Efforts by study cohort to ensure complete 24-hour urine collections.
