# Supplementary material for: Population-Based Limits of Urine Creatinine Excretion
Source: Kidney Int Rep. 2022 Aug 31;7(11):2474–83. doi: 10.1016/j.ekir.2022.08.025 (PMC9751685; doi:10.1016/j.ekir.2022.08.025)

POPULATION-BASED LIMITS OF URINE CREATININE EXCRETION SUPPLEMENTARY MATERIALS

SUPPLEMENTARY TABLES

Supplementary Table S1. Calibration of Jackson Heart Study (JHS) and Chronic Renal Insufficiency Cohort (CRIC) study urine creatinine measurements to isotope dilution mass-spectroscopy (IDMS) standards.
The formulas represent the best fitting linear regression equation.

Description	Formula	R^2
CRIC to IDMS standards	Y = 0.914 * X + 2.557	0.90
JHS to Beckman UniCel DxC 600	Y = 1.071 * X + 1.342	0.96
Beckman UniCel DxC 600 to IDMS standards	Y = 0.802 * X + 3.620	0.99

CRIC: we recalibrated urine creatinine measurements in the CRIC study to isotope dilution mass-spectrometry (IDMS) standards by re-measuring 74 previously collected CRIC urine samples on a Beckman AU5812 analyzer, which is standardized to National Institutes of Standards and Technology (NIST) SRM 3667. The best fitting linear equation relating the original and IDMS calibrated CRIC urine creatinine measurements had an R^2 value of 0.90.

JHS: we recalibrated urine creatinine measurements using a subset of 250 samples and a two-step process. First, we ran samples using a Beckman UniCel DxC 600 analyzer, which resulted in a best fitting linear equation with an R^2 of 0.96. We then applied this equation to the original JHS measurements to derive fitted values and calibrated the fitted values to IDMS standards with a linear equation previously developed using an IDMS gold standard that has an R^2 of 0.99.

Supplementary Table S2. Efforts by study cohort to ensure complete 24-hour urine collections.

Study cohort	Details
CRIC	Written instructions were provided. Samples were considered inadequate if the total volume is less than 500 ml, the total collection time is less than 23 hours or the total urine creatinine test is less than 7 mg/kg of body weight and then a new sample was attempted.
MDRD	Study coordinators ascertained whether collections were complete and accurate. If deemed incomplete, sample was not used and another sample was collected if possible.
JHS	Staff provided written Instructions to participants about how to collect 24-hour urine samples. Staff then verbally instructed participants until it was evident that procedures were clearly understand and emphasized providing complete collections. Participants were provided with one 3-liter collection jar that included a space to write the start and ending times of the collection along with a question that asks if the participant always used the jar during the 24-hour collection. Participants were allowed to indicate a preference for day of collection to minimize problems collecting in the workplace. Prior to the beginning of collection staff contacted participants again to verbally review the instructions. After completion of the collection, staff carefully questioned participants to determine if the collection was complete. If not complete, the samples were not used and the collection was repeated.
PREVEND	Participants received verbal and written information on how to collect 24-hour urine samples. They were instructed to void the bladder in the toilet at the moment of start and to write down this time and to after that add all produced urine into a container until the same time at the next day was reached and to then empty the bladder and add the produced urine to the container and to stop the collection after that time point

CRIC = Chronic Renal Insufficiency Cohort study; MDRD = Modification of Diet in Renal Disease study;

JHS = Jackson Heart Study; PREVEND = Prevention of Renal and Vascular End Stage Disease study.

Supplementary Figure S1. 24-hour urine creatine excretion for women (Figure S1.A) and men (Figure S1.B) by weight, for women (Figure S1.C) and men (Figure S1.D) by height and women (Figure S1.E) and men (Figure S1.F) by age in the discovery cohort. The Cockcroft-Gault equations for weight use the observed average age of 52 years and for age use the observed average weight of 76 kg for women and 88 kg for men.

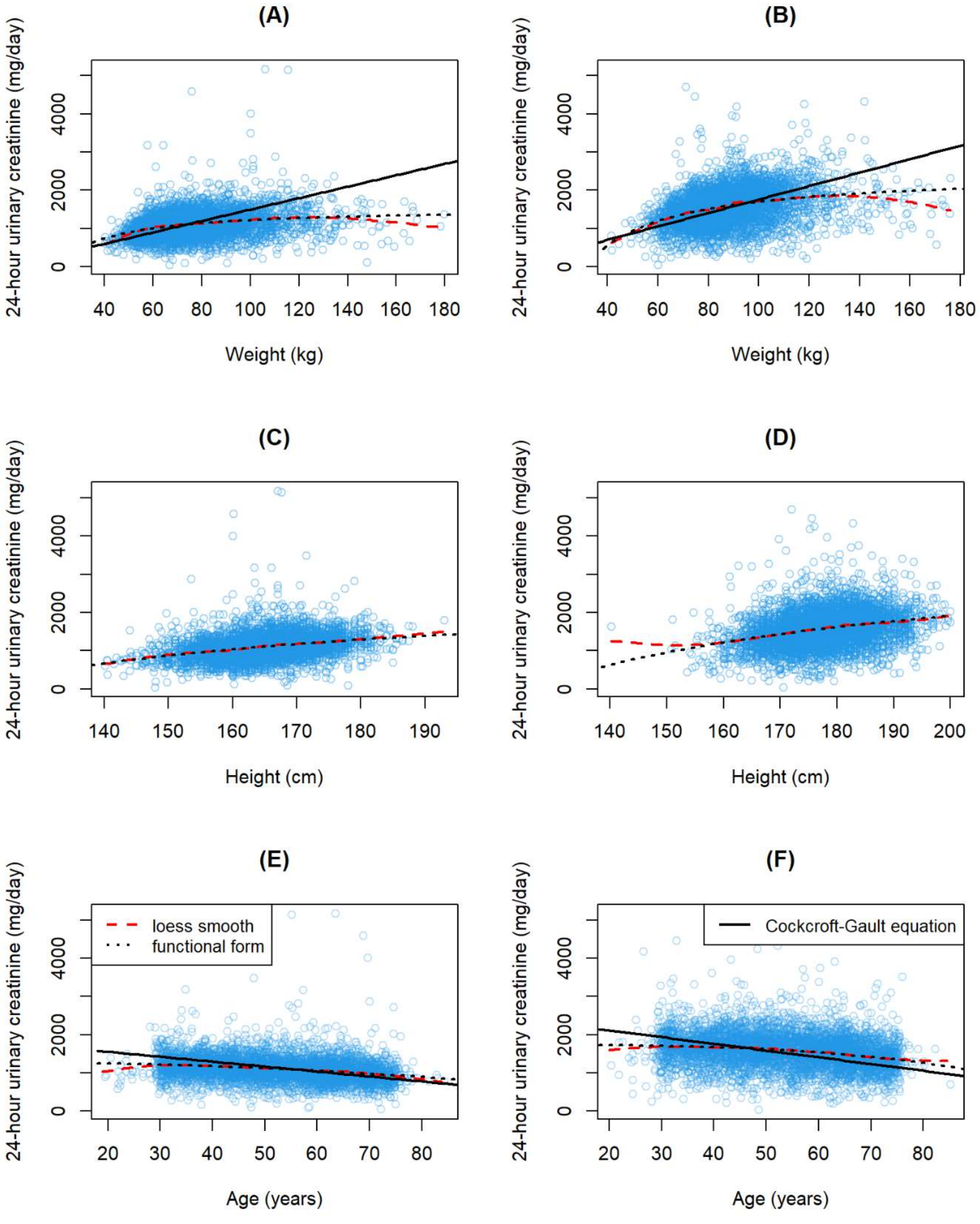

Supplementary Figure S2. Association of serum creatinine concentrations with 24-hour urine creatinine excretion in the discovery cohort for women (Figure S2.A) and men (Figure S2.B).

Y-axis depicts the 24-hour urine creatinine excretion in grams/day and X-axis depicts the serum creatinine concentrations in mg/dL among 9,199 participants in the discovery cohort. Plotting characters represent data from individual participants; solid lines represent sex-specific natural splines with knots located at 0.7 for women, 0.9 for men and at the median and 75th percentile within each sex.

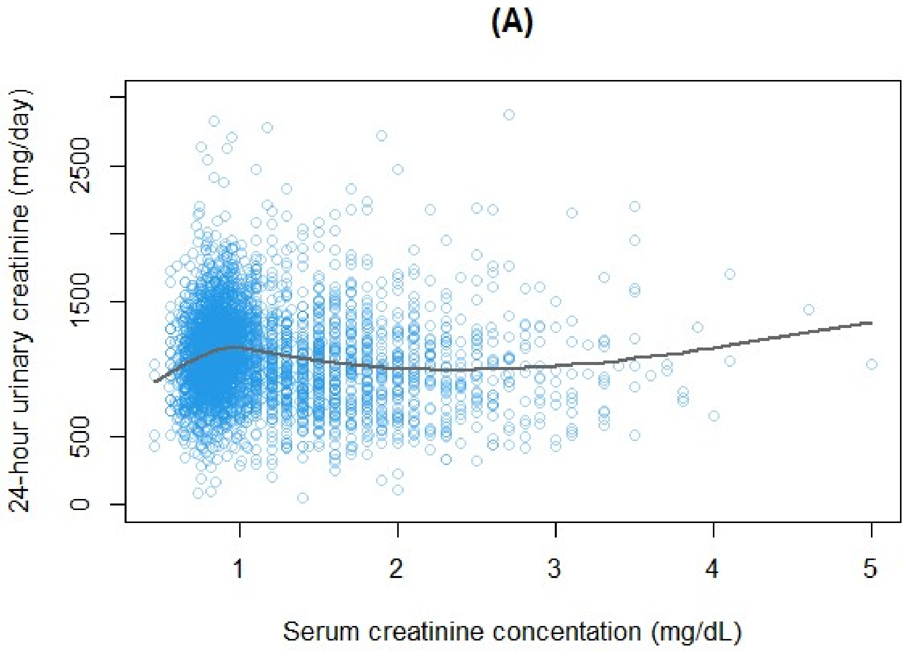

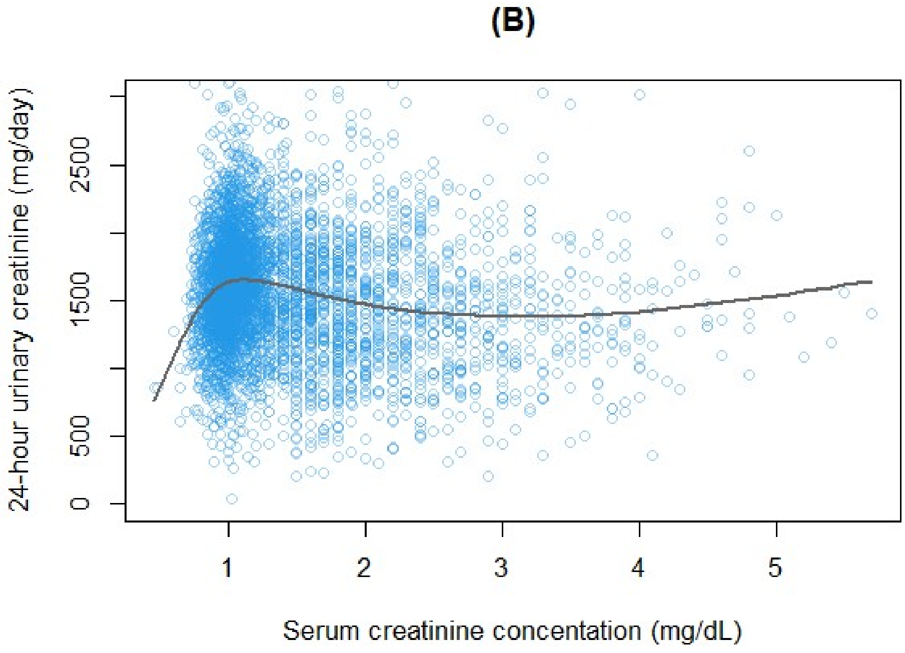

Supplementary Figure S3. 24-hour creatinine clearance and iothalamate clearance measurements of GFR among replication cohort members who completed iothalamate testing in the discovery cohort for women (Figure S3.A) and men (Figure S3.B) and in replication cohort for women (Figure S3.C) and men (Figure S3.D).

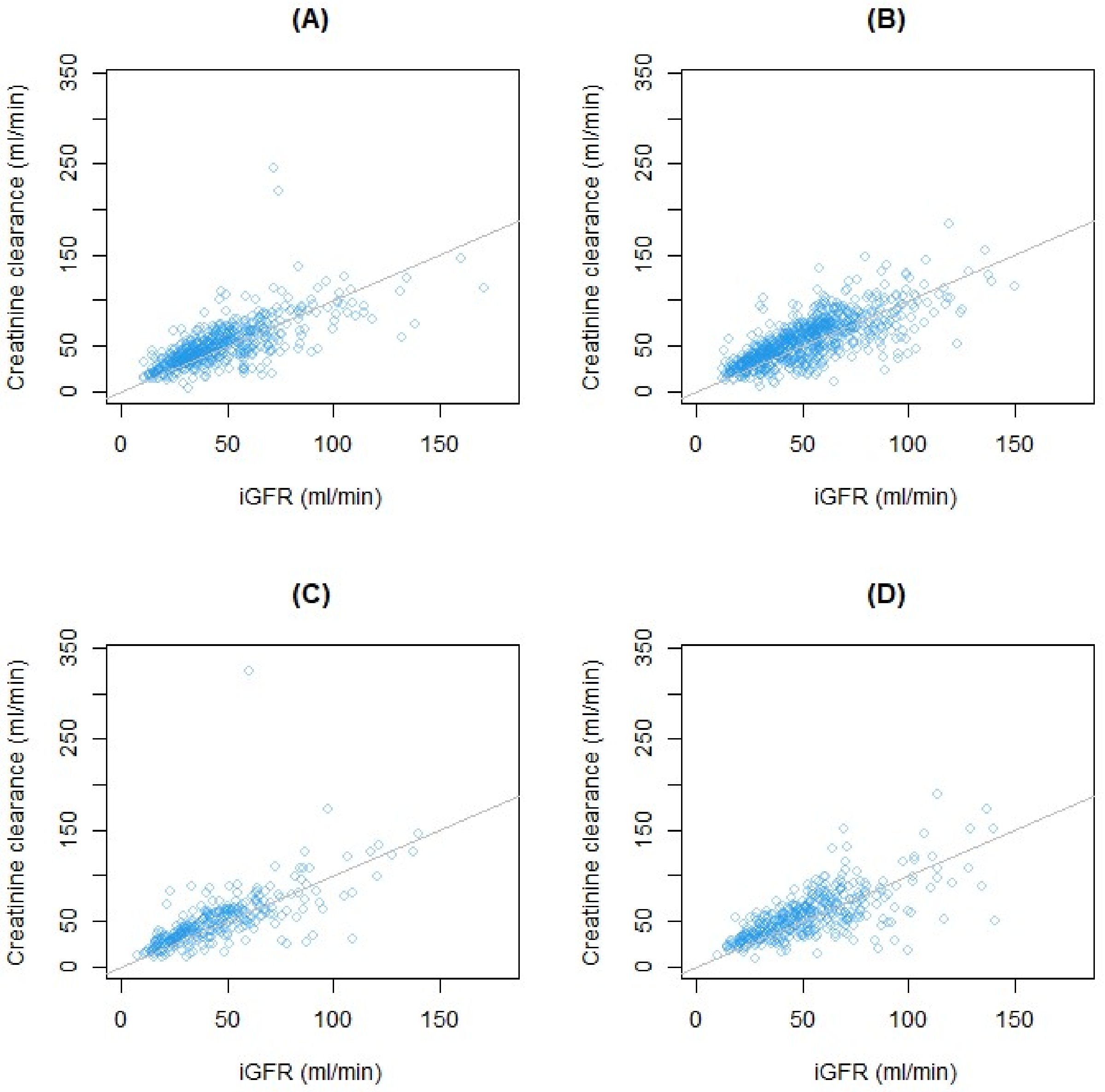

Supplementary Figure S4. Agreement between 24-hour creatinine clearance and iothalamate clearance measurements of GFR among replication cohort members who completed iothalamate testing.

The Y-axis represents the root mean squared error for agreement between creatinine clearance and iothalamate measurements of GFR (lower values indicate greater agreement). The X-axis represents the percent of the replication cohort members selected based on prediction intervals derived from the anthropometric and demographic model. The black line represents the effect of decreasing the lower bound of a two-sided prediction interval, while holding the upper bound at 97.5%.

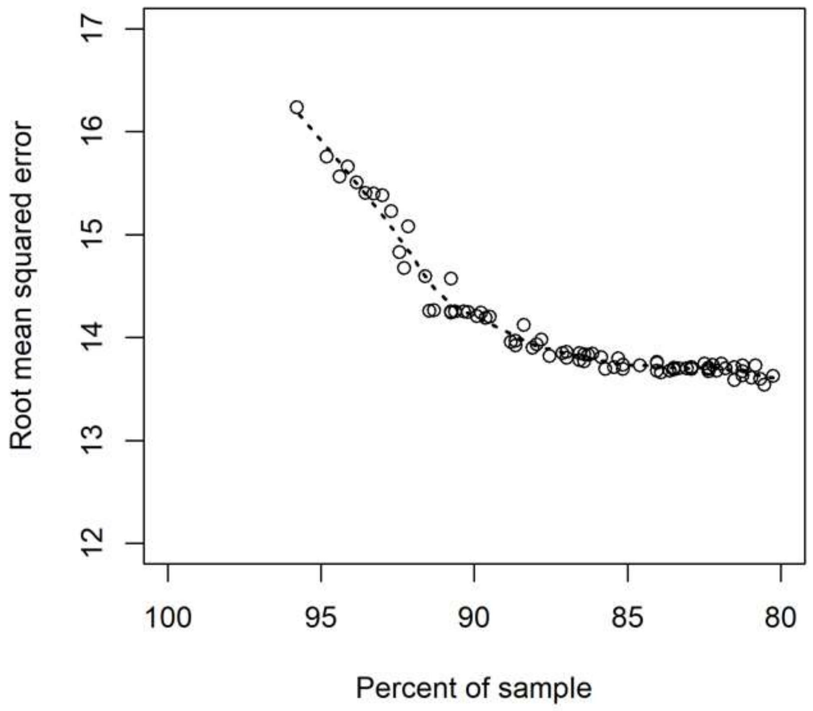

Supplement: Supplementary File (PDF) [file mmc1.pdf]
